# UV-induced damage to DNA: effect of cytosine methylation on pyrimidine dimerization

**DOI:** 10.1038/sigtrans.2017.21

**Published:** 2017-06-09

**Authors:** Lara Martinez-Fernandez, Akos Banyasz, Luciana Esposito, Dimitra Markovitsi, Roberto Improta

**Affiliations:** 1 Istituto di Biostrutture e Bioimmagini, CNR, Napoli, Italy; 2 LIDYL, CEA, CNRS, Université Paris-Saclay, Gif-sur-Yvette, France

## Abstract

Methylation/demethylation of cytosine plays an important role in epigenetic signaling, the reversibility of epigenetic modifications offering important opportunities for targeted therapies. Actually, methylated sites have been correlated with mutational hotspots detected in skin cancers. The present brief review discusses the physicochemical parameters underlying the specific ultraviolet-induced reactivity of methylated cytosine. It focuses on dimerization reactions giving rise to cyclobutane pyrimidine dimers and pyrimidine (6–4) pyrimidone adducts. According to recent studies, four conformational and electronic factors that are affected by cytosine methylation may control these reactions: the red-shift of the absorption spectrum, the lengthening of the excited state lifetime, changes in the sugar puckering modifying the stacking between reactive pyrimidines and an increase in the rigidity of duplexes favoring excitation energy transfer toward methylated pyrimidines.

## Introduction

**Figure 1:**
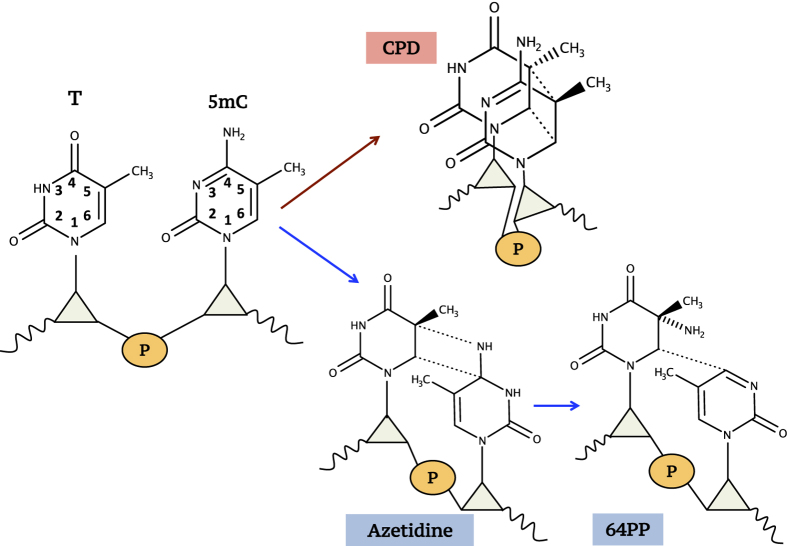
Formation of T5mC dimeric photoproducts. CPD, cyclobutane pyrimidine dimers (*c,s* stereo isomers); 64PP, pyrimidine (6-4) pyrimidone photoproducts, sugars are represented by triangles, phosphate groups by a circle.

Methylation occurring at the 5 position of cytosine (C5-methylation; Figure 1) plays a key role in epigenetic mechanisms involved in the regulation of a variety of biological processes ranging from cell differentiation to gene expression. DNA methylation is also one of the most extensively studied epigenetic modifications in cancer.^1^ As the degree of methylation varies during the cell cycle, 5-methylcytosine (5mC) is considered as the fifth ‘dynamic’ letter of the genetic code.^2–4^ The functions involving 5mC,^5^ and the associated signal transduction, may be highly perturbed by chemical alteration of cytosines provoked by ultraviolet (UV) radiation. In contrast to genetic changes, epigenetic modifications are frequently reversible, which provides opportunities for targeted treatment using specific inhibitors. In this respect, the specificity of 5mC lies in the fact that, although it represents at most 5% of the bases in the human genome, it has been described as an endogenous mutagen.^6,7^ CpG sites contribute to ~35% of all point mutations in the germline ^8^ and are important hotspots for acquired somatic mutations leading to cancer.^6–13^ The degree of correlation between methylation and cancer depends on the tumor type.^6^ In particular, recent studies show that ~40% of melanomas are connected with C5-methylation.^12^ It is well known that 5mC is a mutable site, involved in inherited diseases and in tumors, because it can undergo spontaneous deamination to thymine.^14,15^ Concerning UV-induced mutations, that is the focus of the present contribution, methylation has been correlated with the formation of cyclobutane pyrimidine dimers (CPDs) (Figure 1) occurring in X5mCG sequences, where X represents a thymine (T) or a cytosine (C).^6,16–18^

In a more general way, CPDs may be formed via a direct or an indirect mechanism.^19^ The former involves absorption of photons directly by DNA, while in the latter photons are absorbed by other molecules present in cell, which subsequently react with the nucleic acids. The indirect mechanism is context dependent, being affected by factors such as metabolism, pollution or drugs. In contrast, the direct mechanism corresponds to an intrinsic property of DNA and deserves particular attention.

UV-induced reactions establishing chemical bonds between neighboring pyrimidines lead mainly to two families of dimers (Figure 1): cyclobutane pyrimidine dimers, which may be formed as *cis-syn* (*c,s* CPDs) and *trans-syn* (*t,s* CPDs) stereoisomers, and pyrimidine (6-4) pyrimidone adducts (64PPs). Both types of photoproducts have been detected in dinucleoside monophosphates when one of the reacting pyrimidines is 5mC.^20^ The resulting CPDs may deaminate giving rise to the corresponding thymine photoproducts.^17,20–22^ Such secondary reactions occurring in DNA sequences in which both T5mC and TT repeats are present render the direct correlation between cytosine methylation and dimerization delicate.

A few studies, performed on model systems, genomic and cellular DNA, investigated how methylation affects reaction yields and/or induction^20,23–25^ and reported that it enhances CPD formation, especially for UVB irradiation. Accordingly, among the numerous studies searching the reasons for the increased UV-induced mutagenicity associated with 5mC, one direction explores the very first steps of the complex cascade of events, intervening between photon absorption by DNA and chemical reactions. Performed *in vitro* or *in silico*, such investigations focus on the elementary processes, trying to describe the fate of the photon energy within DNA.

The purpose of the present article is to provide a critical review of various fundamental physicochemical aspects involved in the intrinsic photoreactivity of methylated DNA, exploiting some very recent contributions that combine computational methods (quantum chemistry and molecular dynamics simulations) with optical spectroscopy.

The first section is dedicated to the effect of C5-methylation on the static and dynamic properties of the 5mC monomer excited state, which could affect its photochemical behavior. Then, after concisely discussing the electronic grounds that control photodimerization reactions, we examine systems with increasing complexity. Starting from small oligonucleotides (dinucleotides and trinucleotides) and going to longer single and double strands, we analyze how conformational and electronic modifications induced by C5-methylation influence the studied reactions. Finally, we try to provide a simple and general picture of the present knowledge on the effect of C5-methylation on the photoactivated reactivity of DNA and discuss the main perspectives in this field.

## 5MC monomer

**Figure 2:**
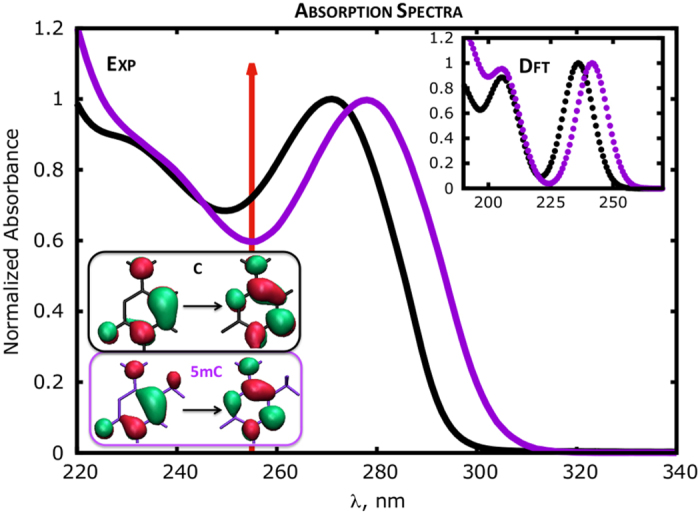
Effect of cytosine methylation on the absorption spectra of nucleosides in water:^30^ dC (black) and 5mC (violet). The red-arrow indicates the irradiation wavelength (255 nm) used in the experiments providing the data reported in Tables 1 and 2. The absorbance of the lower absorption peak is set to 1. Inset: computed TDDFT absorption spectra.^26^

As shown in Figure 2, C5-methylation leads to a noticeable red-shift (*ca.* 10 nm) of the absorption maximum of the nucleosides. For both nucleosides dC and 5mC, this band arises from an electronic transition with a predominant HOMO→LUMO character, that is, involving the excitation of an electron from the highest occupied molecular orbital (HOMO) to the lowest unoccupied molecular orbital (LUMO; Figure 2). This result has been rationalized by recent calculations^26^ showing that the 5-methyl substituent provides an antibonding contribution to the HOMO (as identified by the out-of-phase combination with respect to the C5=C6 π bond), decreasing its stability and, therefore, the HOMO/LUMO gap. The red shift of the cytosine absorption spectrum upon methylation has been considered to be the cause of the increased photoreactivity of 5mC observed in cells.^27^

In addition to the spectral modification, C5-methylation also leads to a noticeable increase of the excited state lifetime. In water solutions, the lowest energy ππ* excited state of dC (S_1_) decays mainly on the sub-ps time scale, whereas the average excited state lifetime of 5mC amounts to several ps.^28–30^ For cytosine, an almost barrierless path on S_1_ leads to a crossing region with the ground electronic state (S_0_), giving account of the very short lifetime and of the very low fluorescence quantum yield. In contrast, in the case of the methylated analog, as discussed in detail in a forthcoming contribution,^31^ the potential energy surface of the lowest excited state contains a minimum, separated from the crossing region with S_0_ by a sizeable energy barrier (0.15–0.3 eV), explaining the increase of the S_1_ lifetime. Since the S_1_ excited state is involved in the photodimerization reaction, as we explain below, a longer lifetime could, in principle, enhance the quantum yield of the reaction. But so far, it has not been possible to assess the significance of this effect on the ground of relevant experimental and computational studies on duplexes.

## Photodimerization: 5MC containing short oligonucleotides

**Figure 3:**
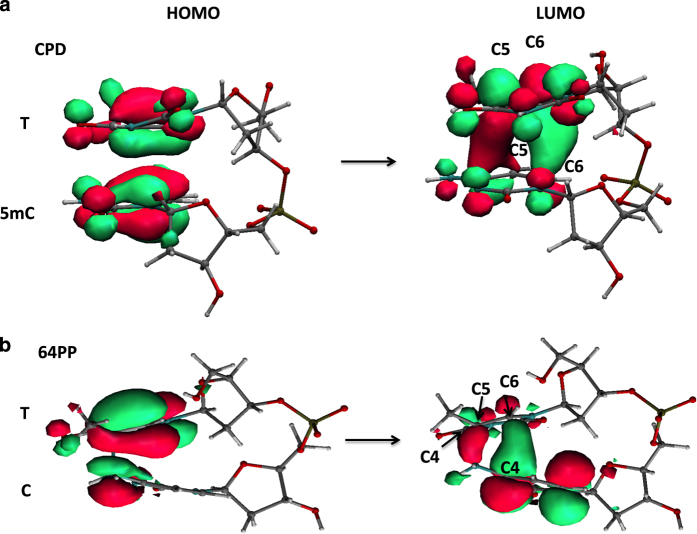
Frontier orbitals involved in the electronic transitions leading to CPD (**a**) and 64PP (**b**) formation.

**Figure 4:**
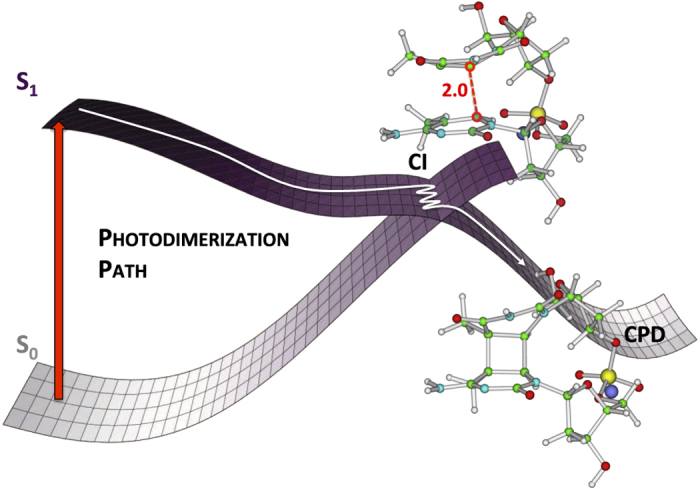
Schematic description of the barrierless path leading to CPD formation.

Before analyzing the effect of C5-methylation on the photodimerization quantum yields, it is useful to provide information on the electronic states involved in these reactions. For what concerns CPD formation, several studies on di-pyrimidine steps (including TC and T5mC steps)^32–40^ show that when two bases are stacked, their S_1_ excited states (the HOMO→LUMO transitions depicted in Figures 2 and 3) interact, giving rise to an electronic transition delocalized over the two pyrimidines. As shown in Figure 3a, this transition involves the excitation of an electron from a molecular orbital (MO) corresponding to the combination of the highest energy π bonding orbitals of the two bases, to a MO deriving from the combination of the two LUMO’s (exciton). Interestingly, this latter orbital has a clear bonding character between the two C5/C5′ and C6/C6′ pairs of the two bases. Light absorption, promoting an electron to this orbital, thus makes the dimerization reaction much easier than in the ground electronic state. Actually, time resolved experimental studies showed that the CPD formation in thymine single strands is ultrafast; the two new bonds are formed essentially within 1 ps after light absorption.^41,42^ In addition, according to quantum mechanical calculations, for certain stacking arrangements of the bases, CPD formation on the S_1_ potential energy surface (PES) is barrierless (Figure 4).^32–40,43^ Consequently, this photodimerization reaction is mainly governed by the ground state conformation: the couple of bases that are in a suitable conformation undergo ultrafast CPD reaction. In particular, it has been proposed that the photo-dimerization is governed by the relative frequency of structures exhibiting short distances between the reactive bonds.^44–46^ More recent contributions also highlight the importance of the sugar puckering adopted by each reactive nucleotide,^32,34^ which affects the stacking geometry of the dipyrimidine steps. Thus, it appears that several conformational parameters are important for CPD formation.

The 64PP formation has been investigated less thoroughly compared to CPDs and mostly for TT steps. There are indications that it proceeds through the so-called oxetane intermediate^47^ followed by 64PP formation on the ms time-scale.^48^ Experiments showed that oxetane formation is faster than 200 ns^48^ but the precise dynamics of the reaction has not been characterized so far. QM studies suggest that the oxetane in the case of TT steps or the azetidine for TC steps are formed on the PES of an electronic state with charge transfer character.^32,33^ The latter can be described as arising from the transfer of an electron from the HOMO of the pyrimidine on the 5′-end toward the LUMO of that on the 3′-end (Figure 3b)^33^ The presence of an energy barrier either in the step leading to the oxetane formation,^33^ or in that leading to the final photoproduct can explain the smaller yield of 64 PP than CPD.^49^

Coming to the experimental results on pyrimidine photodimerization in short oligomers, the first study, performed on 1994 for 5mC-containing dinucleoside monophosphates,^20^ noticed that C5-methylation enhances CPD formation. Two decades later, dimerization quantum yields *ϕ* (and not simple yields), which are necessary for the assessment of the intrinsic reactivity of 5mC, were reported for trinucleotides containing the biologically relevant sequence T5mC and compared to those observed for the non-methylated analogs TCG.^32^ As in the case of the early study, trinucleotides were irradiated at 255 nm. The quantum yields determined for both CPDs (*ϕ*_CPD_) and 64PPs (*ϕ*_64_) are shown in Table 1. In line with what was found for dinucleotides, a higher *ϕ*_CPD_ was determined for T5mCG compared to TCG.

According to its definition, the quantum yield equals to the number of formed dimers divided by the number of absorbed photons. Thus, in order to check to what extent the observed variations of quantum yields arise from the modification of the absorption spectrum of cytosine upon methylation, its contribution can be quantified by the following new parameter:^32^ *I*_m_=*ϕ*_m_ *ψ*_n_/*ϕ*_n_ *ψ*_m_, where *ϕ*_m_ and *ϕ*_n_ are the quantum yields found for the methylated and the corresponding non-methylated system, respectively (Table 2). We remark that the *I*_m_ values determined for *c*,*s* CPDs and 64PPs, 1.7 and 0.8, respectively, being significantly different than 1, reveal that other factors independent from the photon absorption, affect also the pyrimidine dimerization. It is worth-noticing that in contrast to C5-methylation, N4-methylation of cytosine in trinucleotides enhances the yield of 64PPs.^51^

Computational analysis, combining quantum mechanical calculations on TC/T5mC dinucleotides and molecular dynamics simulations on TCG/T5mCG trinucleotides, shows indeed that C5-methylation induces weak, but noticeable structural effects, modulating the conformational equilibria of the dipyrimidine steps. For example, the C2′endo-C1′exo (c2c1) conformer is more stable than the C2′endo-C2′endo (c2c2) one for T5mC, while the opposite is found for TC.^32^ In general, C5-methylation induces a decrease of the pseudo-rotation phase angle, measuring the sugar ring puckering.^32^ Excited state QM calculations show that, independently of C5-methylation, c2c2 conformations are not reactive, since the favored decay path involves localization of the excitation on a single base, followed by ultrafast decay to S_0_ (monomer like decay pathway). On the opposite, for c2c1 arrangement a barrierless path on S_1_ leads to CPD formation. Shortly, C5-methylation favors puckering combinations and, thus, stacking arrangements, that are favorable to CPD formation but less prone to 64PP formation. The role of C5-methylation in modulating the relative importance of ‘monomer like’ and ‘dimerization’ paths has been highlighted in QM studies of other dipyrimidine sequences.^40^

## Longer sequences containing 5MC

Mitchel determined the induction time of CPDs and 64PPs in various model duplexes and concluded that C5-methylation enhances the formation of both types of dimers.^23^ However, it is not possible to correlate the induction time, which is a phenomenological parameter, with physico-chemical factors underlying the reaction mechanisms. Moreover, the conclusions regarding the 64PP enhancement contrasts with an *in vivo* study.^52^

Only one study reports the effect of C5-methylation on the CPD yields in naked human genomic DNA in solution.^24^ Performed *in vitro*, under conditions where solely the direct mechanism is operative and discriminating clearly dimers arising from methylated/non-methylated cytosines, this investigation revealed an interesting point: upon 254 nm irradiation the same CPD yield was observed for methylated and non-methylated DNA, although at this wavelength the molar absorption coefficient of dC is 30% higher than that 5mC.

The above point was further investigated using suitably tailored oligonucleotides: single strands (T5mCGTA)_3_ and (TCGTA)_3_ and duplexes (T5mCGTA)_3_·(TACGA)_3_ and (TCGTA)_3_·(TACGA)_3_.^50^ Their sequence was chosen in a way that pyrimidine dimers arise only from sites that can be methylated so that to avoid confusion with dimers arising from TT sites. The most striking finding is that the *I*_m_ (Table 2) value (representing the change in the reactivity of cytosine upon methylation) determined for duplexes is higher at the shorter wavelength; it is 2.4 at 255 nm and only 1.3 at 282 nm. In contrast, in the case of single strands, although the irradiation wavelength affects *ϕ*_CPD_, it does not have a noticeable effect on *I*_m_. Thus, the observation on duplexes demonstrate, in a more pronounced way than for short systems and longer single strands, that several other factors are responsible for the modification of dimerization efficiency following C5-methylation. From the conformational point of view, molecular dynamics simulations have been applied to DNA fragments with a different extent of methylation (either fully methylated or hemimethylated) and with different sequences (either with repetitive CG sequences, or segments containing interdispersed CG sequences),^53–57^ focusing mainly on CG steps. All these studies agree that C5-methylation decreases the DNA flexibility.

For what concerns the TCG containing long sequences, whose photodimerization quantum yields and *I*_m_ values are also reported in Tables 1 and 2, respectively, MD simulations have shown that the trends found in trinucleotides are maintained in longer single-stranded stretches.^32,50^ In particular, C5-methylation destabilizes the stacking of CG step, favors cytidine C1′exo versus C2′endo conformers, giving rise to a larger population of molecules with short distances between reactive bonds involved in CPD formation. On the other hand, though the average structural features of duplexes are less impacted by C5-methylation, the amplitude of conformational motions is significantly smaller in methylated structures, confirming the results obtained on other sequences.^53–57^ In particular, the increased rigidity of the 5mC-containing duplexes is also revealed by the lower s.d. of the distribution of the distances between the ‘photo-reactive’ bonds.^32^

Quantum mechanical calculations on fragments of these duplexes show that the UV absorption populates excited states delocalized over two or more bases.^50^ The electronic transitions contributing to the maximum of the experimental absorption peak (around 260 nm) are significantly coupled with potentially photochemically active excited states, delocalized over bis-pyrimidine steps. There are thus hints that energy transfer takes place; internal conversion among exciton states (intraband scattering) leads to the bottom of the exciton band, as found experimentally for several nonmethylated DNA duplexes and G-quadruplexes.^58,59^ C5-methylation can enhance the efficiency of intraband scattering, either by increasing the rigidity of the duplex structure (as discussed above), or by decreasing the energy of the lowest energy excited states in reactive dipyrimidine steps.

## Concluding remarks and perspectives

**Figure 5:**
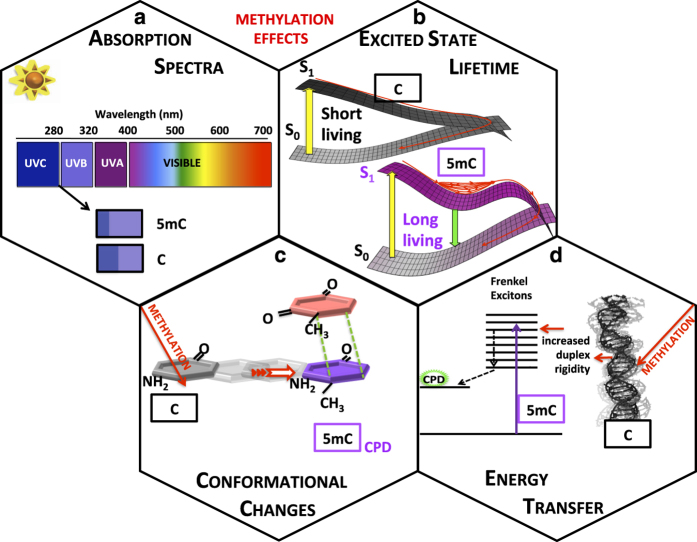
Synoptic scheme summarizing the most significant factors accounting for the effect for C5-methylation on pyrimidine dimerization.

In this contribution, we have provided a general picture regarding the effect of C5-methylation on pyrimidine dimerization and discussed the various physico-chemical factors that may underlie this effect. These factors are schematically illustrated in Figure 5. It is clear that the experimentally observed effects cannot be explained by a single cause and that a subtle interplay among various factors governs the reactivity of methylated DNA.

The red shift of the absorption spectrum induced by C5-methylation (Figure 5a), due to energy destabilization of the HOMO orbital, is certainly an important factor intervening in both dimerization reactions, but it is not the only factor into play. The lengthening of the excited state lifetime (Figure 5b) could contribute to CPD enhancement, but so far there are no available experimental results on duplexes supporting this point. For all types of methylated systems, structural effects, favoring conformations more reactive toward CPD formation (Figure 5c) and modulating the role of the flanking bases,^60,61^ are certainly a key factor. Finally, C5-methylation increases the duplex rigidity (Figure 5d), facilitating the energy transfer from non-reactive bases to reactive ones, via delocalized excited states (excitons).

The role of the above-mentioned factors is more easily assessed in the case of CPDs, whose formation takes place in a single step via delocalized excited states.

As 64PP formation takes place via a two-step mechanism (the first involving a charge transfer excited state between the two reactive pyrimidines) and the experimental observations concern so far the overall reaction, the direct correlation of calculated parameters with experimental observations is delicate.

Notwithstanding the significant advances made since the beginning of the 21st century in our understanding of how DNA methylation affects pyrimidine dimerization caused by direct absorption of UV radiation, several important issues remain to be elucidated.

Starting from photon absorption, it would be interesting to explore the absorption of methylated systems in the UVA spectral domain. In the case of non-methylated systems, it was shown recently that, in contrast to the isolated bases, both naked genomic DNA and model duplexes absorb in this spectral domain. This absorption, giving rise to CPDs,^62,63^ was correlated to charge transfer transitions among reactive pyrimidines,^34^ which may be vibronically coupled to ππ* transitions.

Regarding the role of the excited state lifetime, ultrafast spectroscopy, probing the IR spectral domain, already used for the study of thymine CPDs, could bring valuable information for the dynamics of dimerization reactions.

The conclusions concerning the role of conformation and energy transfer were drawn from joint experimental and theoretical investigations on systems containing the sequence TCG/T5mCG. Similar studies on other sequences, as for example CCG/C5mCG, would allow checking in which extent these conclusions have a general validity.

## Figures and Tables

**Table 1:** Quantum yields *ϕ* (×10^3^) determined for the formation of dimeric photoproducts following irradiation at 255 nm

	*TCG* ^32^	*T5mCG* ^32^	*n-ss* ^50^	*m-ss* ^50^	*n-ds* ^50^	*m-ds* ^50^
*ϕ*_CPD_ (*c*,*s*)	0.5	0.7	1.3	1.3	0.6	1.1
*ϕ*_64_	0.6	0.4	1.4	0.3	0.7	0.1

Abbreviations: CPD, cyclobutane pyrimidine dimer; 64, pyrimidine (6-4) pyrimidone adducts; ds, double strand; m, methylated; n, non-methylated; ss, single strand.

**Table 2:** Quantification of the effect of C5-methylation on the pyrimidine dimerization following irradiation at 255 nm by the parameter *I*_m_=*ϕ*_m_ *ψ*_n_/*ϕ*_n_ *ψ*_m_ accounting for the different absorption spectra of C and 5mC; *ϕ*_m_ and *ϕ*_n_ are the quantum yields found for the methylated and the corresponding non-methylated system, respectively (Table 1); *ψ*_m_ and *ψ*_n_ represent the fraction of photons absorbed by a reactive 5mC or C

	*Trinucleo tides* ^32^	*Single strands* ^50^	*Double strands* ^50^
CPDs (*c*,*s*)	1.7	1.3	2.4
64PPs	0.8	0.3	0.3

Abbreviations: CPD, cyclobutane pyrimidine dimer; 64, pyrimidine (6-4) pyrimidone adducts.
